# Research models and mesenchymal/epithelial plasticity of osteosarcoma

**DOI:** 10.1186/s13578-021-00600-w

**Published:** 2021-05-22

**Authors:** Xiaobin Yu, Jason T. Yustein, Jianming Xu

**Affiliations:** 1 Department of Molecular and Cellular Biology, Baylor College of Medicine, One Baylor Plaza, Houston, TX 77030 USA; 2 Department of Pediatrics, Texas Children’s Cancer and Hematology Center, and The Faris D. Virani Ewing Sarcoma Center, Baylor College of Medicine, One Baylor Plaza, Houston, TX 77030 USA

**Keywords:** Osteosarcoma, Metastasis, Experimental model, EMT/MET-related process, EMT-TFs

## Abstract

Most osteosarcomas (OSs) develop from mesenchymal cells at the bone with abnormal growth in young patients. OS has an annual incidence of 3.4 per million people and a 60–70% 5-year surviving rate. About 20% of OS patients have metastasis at diagnosis, and only 27% of patients with metastatic OS survive longer than 5 years. Mutation of tumor suppressors RB1, TP53, REQL4 and INK4a and/or deregulation of PI3K/mTOR, TGFβ, RANKL/NF-κB and IGF pathways have been linked to OS development. However, the agents targeting these pathways have yielded disappointing clinical outcomes. Surgery and chemotherapy remain the main treatments of OS. Recurrent and metastatic OSs are commonly resistant to these therapies. Spontaneous canine models, carcinogen-induced rodent models, transgenic mouse models, human patient-derived xenograft models, and cell lines from animal and human OSs have been developed for studying the initiation, growth and progression of OS and testing candidate drugs of OS. The cell plasticity regulated by epithelial-to-mesenchymal transition transcription factors (EMT-TFs) such as TWIST1, SNAIL, SLUG, ZEB1 and ZEB2 plays an important role in maintenance of the mesenchymal status and promotion of cell invasion and metastasis of OS cells. Multiple microRNAs including miR-30/9/23b/29c/194/200, proteins including SYT-SSX1/2 fusion proteins and OVOL2, and other factors that inhibit AMF/PGI and LRP5 can suppress either the expression or activity of EMT-TFs to increase epithelial features and inhibit OS metastasis. Further understanding of the molecular mechanisms that regulate OS cell plasticity should provide potential targets and therapeutic strategies for improving OS treatment.

## Background

Osteosarcoma (OS) is the most common type of cancer that initiates at the bone, with a worldwide incidence of 3.4 per million people each year [1]. The 5-year survival rate for classic OS was only 20% during most of the twentieth century until the introduction of adjuvant chemotherapy in the 1970s [2]. The routine treatment of high-grade OS also shifted from amputation to chemotherapy and limb salvage by 1990, with a subsequent increase in overall survival rate to more than 65% [3, 4].

**Fig. 1:**
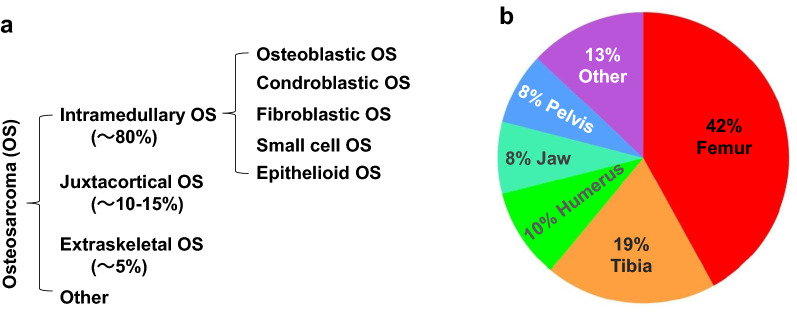
The types and subtypes (**a**) as well as the skeletal distribution (**b**) of osteosarcomas

OS arises from primitively transformed cells with a mesenchymal origin [4]. The cancer cells in OS look like early forms of bone cells that normally help generate new bone tissues, but the bone tissues in OS are not as strong as that of normal bones [5]. Each year, about 800 to 900 new cases of OS are diagnosed in the United States [5]. Based on patient ages and causes of OS development, they can be classified into primary and secondary OSs. The primary OS typically develops in young patients as a result of abnormal bone development. The secondary OS occurs in patients over 65 years old, and is usually secondary to malignant conditions of Paget’s disease, post-irradiation exposure, severe bone infarct, osteochondroma and osteoblastoma [6]. Based on the location and appearance, OSs can be classified into intramedullary, juxtacortical, and extraskeletal OSs, and each of these OS types can be further divided into several subtypes [6]. Intramedullary OS is the most common and the fastest growing type, which accounts for nearly 80% of all OSs. Intramedullary OS develops in the medullary cavity of a long bone, such as femur and tibia. Furthermore, there are a number of subtypes of intramedullary OS according to the tumor cell types. The common subtypes include osteoblastic, chondroblastic, fibroblastic, small-cell, and epithelioid OSs. Juxtacortical OS is the second most common type, and they account for 10 to 15% of all OSs. This type of OS develops on the outer surface of the bone, or periosteum that is the dense layer of connective tissue covering the bone. Extraskeletal OS is rare and grows slowly, accounting for fewer than 5% of all OSs. Extraskeletal OS does not touch the bone and often arises from soft tissues that have experienced prior radiation therapy (Fig. 1a).

Most OSs occur in children and young adults at ages from 10 to 30 years old, with the peak incidence in the second decade of life; however, people at any ages can develop OS [7]. OS is the third most common cancer in adolescence, accounting for approximately 3% of all adolescent cancers, with only lymphoma and brain tumor being more prevalent [1]. Adolescent OS usually develops in a region where the bone grows rapidly, such as the end region of the long leg or arm bones [8]. In some cases, OS occurs in the humerus, ulna, radius, fibula or pelvis (Fig. 1b) [9]. OS occurs slightly more common in boys versus girls, which may be related to the taller body height correlated with faster bone growth in boys on average. However, girls tend to develop OS slightly earlier, which may be related to their earlier growth spurt on average. The incidence of OS is higher in black population compared with white and other populations [10]. In the United States, more than 50% of all OS arising in patients over the age of 60 are the secondary OSs. Unlike OSs in children and young patients, OSs in elder patients more commonly develop at axial locations and in tissue areas that have previously received irradiation or have existing bone abnormalities [11]. OS patients older than 60 years are associated with a higher risk of metastatic disease [8]. Elder men are also associated with higher risks to develop OS than elder women do. In contrast to the OS disparity in black children, OS is more frequently observed in white elder adults compared with black and other elder adults [10]. The incidences of OS among 24-year-old and younger individuals are generally consistent in different countries in the world, with most cases diagnosed during puberty. However, the incidences of OS among elder men are higher in some countries including United Kingdom, Australia, and Canada compared with other countries [12].

Most OSs occur sporadically, and the exact causes for OS development are still not fully understood. Especially, the vast majority of OS cases in adolescents and young adults are sporadic with no known familial genetic or environmental causes [13–15]. However, it has been noticed that most OS cases occur in patients with certain rare inherited types of cancers or diseases such as retinoblastoma, Li–Fraumeni syndrome, and Rothmund–Thomson syndrome, which involve chromosomal abnormalities of the tumor-suppressors *RB1* and *TP53*, as well as the DNA helicase *REQL4* genes, respectively [6, 11, 16]. Individuals carrying germline *RB1* mutations have approximately 1000-fold increased risk to develop OS [17]. Abnormalities in the *CDKN2A* gene, which codes for p16INK4a, a CDK4 inhibitor, and p14ARF, a p53 stabilizer, also increase the risk of OS development [6]. OS occurs more commonly at the regions of bone growth, which is presumably attributed to the genomic mutations acquired from the active cell proliferation. People receiving radiation therapies for treating other types of cancers may have a higher risk to develop OS later from the treated tissue sites. Indeed, people treated with radiotherapies at younger ages or received high irradiation doses have increased risks to develop OS [11]. In addition, people with certain non-cancerous bone diseases such as Paget’s disease and hereditary multiple osteochondromas also have increased risks to develop OS. Specifically, about 1% of Paget’s disease patients actually develop OS [11].

About 20% of OS have disseminated to the lung, brain or other organs at the time of diagnosis [18]. Chemotherapy and surgery are the most common treatments for most OS patients [1, 19]. Chemotherapies given before and/or after the surgery significantly reduce the risk of recurrence. The most commonly used chemotherapy drugs include methotrexate, doxorubicin and cisplatin, and two or more of these drugs are usually administrated in combination [19]. The 5-year survival rate of all-stage OSs is about 60%. However, if metastasis has happened at the time of diagnosis, the 5-year survival rate drops significantly to only 27%, which has not been improved significantly over the last four decades [16]. Recurrent or metastatic OSs are usually resistant to currently available standard treatments. Therefore, it is necessary to understand the detailed mechanisms responsible for OS development, progression and metastasis in order to identify novel therapeutic targets for treating OS.

## Cellular and animal models for studying OS

### Canine OS models

Spontaneous OSs are more common in large dogs compared with humans, making dog an attractive model to study this disease [20]. Canine OS is similar to that of human OS in terms of biological features and clinical symptoms [21, 22]. It is estimated that over 10,000 cases of canine OS occur annually in the United States. The major difference between canine OS and human OS is that canine OS is a disease of large breed elder dogs (6–12 years of age), which is considered as a limitation to use canine OS models to study human OS. The median disease-free intervals are 4 months after single surgery treatment, and 13 months after combined treatment of surgery and chemotherapy [23–25]. Many genomic alterations involved in human OS pathogenesis are also detected in canine OSs, such as the loss-of-function genetic alterations of the *TP53* [26–28], *RB* [29] and *PTEN* [30] tumor suppressor genes in both human and canine OSs.

### Radiation or chemical carcinogen induced OS models

Historically, rodent OS models began with the exposure of rats or mice to chemical and radioactive carcinogens [31, 32]. Murine OS models have been induced by exposing animals to radioactive substances such as radium, thorium and roentgen [33]. Despite their high incidences, these models probably represent secondary OSs developed in human patients who have received previous irradiation treatment, which might not share molecular mechanisms with primary OSs. Most of the chemically induced mouse OS models have been developed by injecting different chemical carcinogens directly into bones [33]. For example, rats treated with P^32^-orthophosphate have been shown to develop OS tumors that histologically resemble human OS [31, 34]. However, the carcinogen-induced murine model is more representative of a therapy-induced disease, while most human OSs are sporadic [35].

### Animal OS-derived cell lines

Along with the rapid development of cancer immunotherapy, cancer cell lines isolated from spontaneously developed animal tumors have become important syngeneic models for studying immune suppression and activation, as well as interactions between immune and cancer cells in the same strain animals with identical genetic background (Table 1). The first batch of successfully established metastatic OS cell lines including K7, K8, K12, K14 and K37 were derived from a spontaneous OS in the distal femur of an 895-day-old female BALB/c mouse [34, 36–40]. These lines were metastatic in vivo and have been used for years to study the process of OS metastasis [41]. The Dunn cell line is another murine OS cell line derived from a spontaneous OS in the tail of a C3H/HeN mouse. This cell line is metastatic in vivo, and its xenograft tumors commonly metastasize to lung and liver in mice [42]. Multiple sublines derived from Dunn cell line have been used as OS angiogenesis and metastasis models in screening new compounds and testing candidate drugs [43–46]. The UMR 106-01 cell line was developed from a ^32^P-induced OS tumor in a Sprague-Dawley rat [32, 47]. This cell line has been well adopted to OS research due to its phenotypical similarities to human OS cells and rapid formation of pulmonary metastasis [48–51]. Besides murine, a number of cell lines have been derived from spontaneous canine OSs. Among these canine OS cell lines, the D-17 cell line was isolated from the lung metastasis of an 11-year-old female poodle. D-17 cells have been widely used in finding therapeutics for bone cancers in dogs [52, 53] (Table 1).

**Table 1:** The origin, morphology, genetic mutation, tumorigenic capability, and metastatic potential of OS cell lines

Cell line	Origin	Morphology	Gene mutation						Days to form 1.5–2 cm^3^ tumor	Meta rate (%)	Refs.
			Tumor suppressor				Oncogene				
			CDKN2A	TP53	RB1	PTEN	BRAF	KRAS			
HOS	OS of a 13-y-o girl	E	m/m	m/m	w/w	w/w	w/w	w/w	No tumor in 8 weeks	0%	[58, 64]
143B	HOS cells, transformed by KRAS	Mixed E/F	m/m	m/m	w/w	w/w	w/w	m/w	27	100%	[64, 69]
MNNG	HOS cells, induced by MNNG	Mixed E/F	m/m	m/m	w/w	w/w	w/w	w/w	32	100%	[64, 66, 70]
SaOS-2	OS of a 11-y-o girl	E	w/w	m/m	m/m	w/w	w/w	w/w	95	100%	[62, 63]
SaOS-LM7	Lung Meta from SaOS-2 cells in mouse tibia	E	w/w	m/m	m/m	w/w	w/w	w/w	65	100%	[71]
MG-63	Fibroblastic OS of a 14-y-o boy	F	m/m	m/m	w/w	w/w	w/w	w/w	170	0%	[59]
U-2 OS	Sarcoma in tibia of a 15-y-o girl	E	w/w	w/w	w/w	w/w	w/w	w/w	No tumor in 150 days	0%	[60, 61, 72]
K7 (K8, …, K12)	Spontaneous OS in BALB/c mouse	O	NA	NA	NA	NA	NA	NA	NA	33.3% (K12)	[37, 38, 45]
K7M2	Lung meta From K7 cells in mouse tibia	O	NA	NA	NA	NA	NA	NA	30 days to reach 0.8 cm^3^	93.3%	[36, 37, 39, 40, 45]
Dunn	Spontaneous OS from C3H/HeN mouse tail	E	NA	NA	NA	NA	NA	NA	25	100%	[42, 45]
UMR 106-01	^32^P-induced tumor in rat	E	NA	NA	NA	NA	NA	NA	25 days to reach 0.15 cm^3^	100%	[32, 47]
D-17	Lung Meta of OS in a 11-y-o female poodle	E	NA	NA	NA	NA	NA	NA	No tumor in 79 days	NA	[53]

m/m, mutant/mutant homozygous; w/w, wild type/wild type normal alleles; m/w, mutant/wild type heterozygous; NA, not available; E, epithelial; F, fibroblast; O, osteoblast; Meta, metastasis

### Human OS-derived cell lines

U2OS, the first human OS cell line, was established in the year of 1964, and has been used extensively in many in vitro studies. One limitation of this cell line is that it does not satisfy researchers’ needs for an in vivo metastatic model [54–61]. Unfortunately, this was also the case for other several subsequently established human OS cell lines such as HOS and SaOS2 cell lines [58, 62, 63]. In 2005, the HOS cells were treated with carcinogen or virus-mediated oncogene expression to induce genetic alterations, and the first metastatic human OS cell line 143B, along with several other derivative cell lines were established from these treated cells [58, 64–71]. Thereafter, many metastatic cell lines have been established in vitro, which have formed the basis for studying the cellular and molecular processes of OS (Table 1). A recent study characterized a set of 19 OS cell lines by profiling their gene expression and epigenetic patterns, and by comparing their differentiation, growth, invasion, and migration capacities in nude mice [72]. These valuable data should facilitate investigators to select appropriate OS cell lines for their researches.

### Genetically engineered OS mouse models

Cell culture models may impose widespread genetic changes and loss of phenotypic heterogeneity that diverge from the characteristics of the original OSs. Taking SaOS2 as an example, OS cells maintained in culture demonstrate significant changes in phenotype over time [73]. In general, higher passage cells exhibit higher proliferation rates and lower alkaline phosphatase activity, while mineralization is more pronounced in late passage cells. Gene expression profiles may also change in culture over time. Genetically engineered mouse (GEM) models may help to provide spontaneously developed OS models with natural tumor environment for studying OS initiation, growth and metastasis.

The first GEM model of OS is the H2K-fos-tg mouse model, where c-fos is overexpressed in osteoblasts to induce OS development. The tumors developed in this model display similar histopathology to human osteoblastic OS, but these tumor cells do not produce distant metastasis that frequently occurs in human OSs [74]. Many murine OS models have been developed to recapitulate p53 and RB mutations in hereditary and sporadic human OSs [75]. Germ-line deletion of p53 results in an OS incidence of 4% in homozygous p53 null mice [76] and 25% in heterozygous p53 mice [77], indicating the importance of p53 loss in OS development. The higher OS incidence in heterozygous versus homozygous p53 knockout mice may be due to the development of other types of cancer that results in early death of the homozygous knockout animals. However, homozygous Rb knockout mice are lethal before birth, and heterozygous Rb knockout mice do not develop OS [78, 79].

The application of conditional gene manipulation and tissue specific Cre expression in mice have greatly enhanced the ability to induce OS from mesenchymal osteogenic cell lineages to model human OSs. Deletion of both *Tp53* and *Rb* genes by Osterix-Cre leads to OS development with high penetrance [80]. Mice with Prx-1-Cre mediated deletion of both *Tp53* and *Rb* genes induces OS development, and also generated poorly differentiated soft tissue sarcomas [81]. Mice with Osterix-Cre activated expression of a transgenic shRNA that targets p53 mRNA also develop osteoblastic OS at 100% penetrance. Although this model exhibits a longer latency to tumor onset, these OSs often develop in long bones and are highly metastatic to lung and liver. More importantly, this model does not develop any non-OS tumors [82].

In addition to c-fos and p53, other proteins such as TWIST1 [83], p14ARF [84], p16INK4a [85], PRKAR1A [86], and p21CIP [87] have been implicated in OS pathogenesis based on findings from human OS samples, and their involvements in OS development have also been demonstrated in GEM models. Their alterations appear to complement the defects in the p53 and Rb pathways. Although these models provide further insights into OS genetics and biology, the long latency combined with low penetrance makes these models less practical.

### OS cell-derived xenograft models

Human or mouse OS cells are routinely inoculated into immunocompromised mice to grow xenografts and allografts as OS models. The injected cells usually develop solid tumors within days or weeks. The advantages of these models include quick onset, affordable cost, ease of handling and maintenance, and high reproducibility. Although OS cells are ectopically inoculated under the skin in some studies, cell grafts that grow orthotopically in or near bones are considered as more relevant preclinical OS models. These OS cell-derived xenograft models have been very useful in identification of factors that are involved in OS invasion and drug candidates that inhibit OS growth [36, 37, 41, 88]. An obvious limitation of these xenograft models is that they do not provide information about the initiation and etiology of OS since it uses fully immortalized OS cells.

### Patient-derived xenograft (PDX) OS models

To establish a PDX line, small pieces of fresh tissue from either an incisional (open) biopsy or a percutaneous (needle) biopsy of an OS tumor in a patient are transplanted into multiple immune-defective mice to grow xenograft tumors [89]. In PDX models, the OS tumor cells are never cultured in vitro and always maintained in an OS-like tissue environment, and thus, PDX models are considered as a much more clinically relevant model to represent both the general features and heterogeneity of human OSs [90, 91]. The PDX models also allow the study of early-stage progression of OS metastasis in vivo. The PDX models have been broadly used for screening and testing drugs for developing new therapies [92–94]. However, like any other models, the PDX models are not perfect. Some initial generations of PDXs may take a long time to form tumors, suggesting that these OS cells still experience senescence, immortalization, and/or growth selection processes before a small population of tumor cells grows to form a tumor. Additionally, along with the tumor growth, most, if not all, of the stromal cells such as fibroblasts, immune cells, vascular cells and fat cells in the tumor environment are replaced with mouse cells, which means that the human OS tumor cells still grow in a mouse tissue and cellular environment.

## The mesenchymal and epithelial plasticity in OS progression

**Fig. 2:**
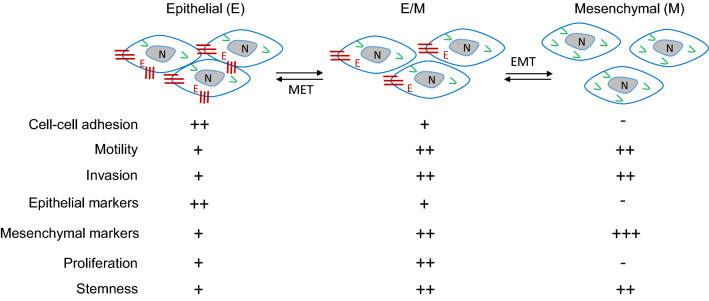
The different cellular features associated with the different plasticity states of osteosarcoma (OS) cells. In this model, OS cells can maintain different states of hybrid epithelial/mesenchymal phenotypes characteristic of different expression levels of epithelial and mesenchymal markers. These OS cells can undergo either more epithelial or more mesenchymal transition states, which are correlated with different cellular features associated with the aggressiveness of OS. EMT, epithelial-to-mesenchymal transition; MET, mesenchymal-to-epithelial transition; V, vimentin; E, E-cadherin; N, nucleus. +++, strong or high; ++, moderate; +, weak; −, negative

Epithelial-to-mesenchymal transition (EMT) and its reverse process, mesenchymal-to-epithelial transition (MET), are required for adapting cell plasticity in many physiological and pathophysiological processes, such as embryonic development, wound healing, fibrosis and cancer metastasis [95]. The cancer cells with an epithelial origin such as breast and prostate adenocarcinoma cells can undergo EMT to acquire mesenchymal gene markers and morphologies. Cancer cells at an EMT state are highly capable to escape from epithelial tumor cell clusters, invade into the stromal tissue, and disseminate to distant organs [96, 97]. Once getting in a distant organ, a MET process is thought to help the disseminated cancer cells to adapt the new tissue environment for proliferation and establishment of metastasis [96]. Cancer cells can have various degrees of EMT or MET statuses, ranging from full epithelial to full mesenchymal states (Fig. 2) [95]. Most sarcoma cells including OS cells exhibit an epithelial or a hybrid mesenchymal and epithelial phenotype. Since sarcoma cells are inherently locked in a mesenchymal state, they unlikely reprogram to a full epithelial state. However, depending on sarcoma’s histiotypes, they can reprogram their degrees of epithelial and mesenchymal states during their growth, progression and metastasis. Indeed, although the features of epithelial and mesenchymal plasticity are variable across OSs with different histiotypes, EMT and MET events are frequently observed and regulated by many molecular players [98].

### EMT-promoting transcription factors (EMT-TFs) in OS and other sarcomas

EMT has been extensively studied in carcinomas in which EMT properties are associated with drug resistance, invasion and metastasis [96]. Sarcoma has a mesenchymal origin, and its mesenchymal phenotype is maintained by the functions of EMT-TFs, including TWIST1, SNAIL, SLUG, ZEB1 and ZEB2, and associated with more aggressive behaviors. Depending on different patient cohorts, variable percentages (32–56%) of human OSs have TWIST1 expression. The TWIST1-positve OS cells are associated with metastatic phase III OS tissues and also with poor clinical outcomes [99, 100]. SNAIL is widely expressed in OSs [101]. Knockdown of SNAIL in SaOS2 cells increases E-cadherin expression to promote MET, which is accompanied with decreased cell migratory and invasive properties. Conversely, overexpression of SNAIL in SaOS2 cells suppresses E-cadherin expression to promote EMT-like process, which promotes invasion and metastasis of these cells [102]. The expression of SLUG is associated with high-grade cranial bone OSs with high metastatic potentials [103]. Knockdown of SLUG in canine D-17 and human SaOS2 cells significantly decreases the migration and invasion capabilities of these OS cells by remodeling their actin cytoskeleton organization and disrupting their cellular protrusions. Knockdown of SLUG also inhibits the growth of these cell-derived xenografts in an in vivo chick chorioallantoic membrane (CAM) assay model. Ectopic expression of SLUG in these OS cells increases the expression level of WNT5A, decreases the expression level of the adhesion molecule osteoblast cadherin (OB-Cad), and increases cell motility by promoting the formation of actin-rich cellular protrusions [104]. The expression levels of ZEB1 protein in human sarcoma tissues are also positively correlated with lung metastasis, which is consistent with the finding showing that ZEB1 knockdown in MG-63 cells significantly inhibits cell invasive capability [105]. The mixed canine benign mammary tumors are composed of epithelial cells and cartilage or bone tissue, which is a species-specific type. The malignant canine mammary tumors include carcinomas, fibrosarcomas and OSs. In comparison with canine mammary epithelial cells and carcinomas, fibrosarcomas express high and OSs express even higher levels of a panel of homeobox genes including ZEB2. ZEB2 is an EMT-TF important for craniofacial bone formation [106]. Collectively, these studies suggest that the EMT-TFs play important roles in maintaining the mesenchymal status of sarcomas. Understanding the molecular mechanisms underlying these EMT-TFs regulated OS cell growth and metastasis may provide new opportunities to identify potential molecular targets for treating OSs.

### MET in OS and other sarcomas

Since sarcomas have a mesenchymal cellular origin, more studies have been carried to understand the role of MET in sarcoma progression. Patients with more epithelial-like carcinomas tend to have better clinical outcomes compared with patients with more mesenchymal-like carcinomas, and a similar trend is also the case for patients with sarcomas. E-cadherin is the first epithelial marker detected in bone and soft tissue sarcomas [107]. A meta-analysis of 812 bone and soft tissue sarcoma tumors demonstrates that low E-cadherin expression is associated with poor five-year overall survival [108]. Ewing sarcoma/primitive neuroectodermal tumor (ES/PNET) cells frequently express epithelial markers such as cytokeratins, claudin-1 and ZO-1, and exhibit a partial epithelial differentiation state. This study showed evidence for expression of tight junction proteins such as claudin-1 and ZO-1 in over 50% of ES/PNET samples, suggesting partial epithelial differentiation in this kind of cancer [109]. It is also reported that patients with leiomyosarcomas that express high epithelial signature genes including E-cadherin also have a better prognosis [110, 111]. In OSs, E-cadherin expression levels have been found to be inversely correlated with metastasis potential but positively correlated with good prognosis [99]. However, it should be noted that the process of MET in sarcoma is characterized by increased expression of epithelial-like markers such as E-cadherin, whereas the typical mesenchymal markers including vimentin remain abundantly expressed in the sarcoma cells [111, 112].

### The signaling pathways and EMT-TFs that regulate MET

**Fig. 3:**
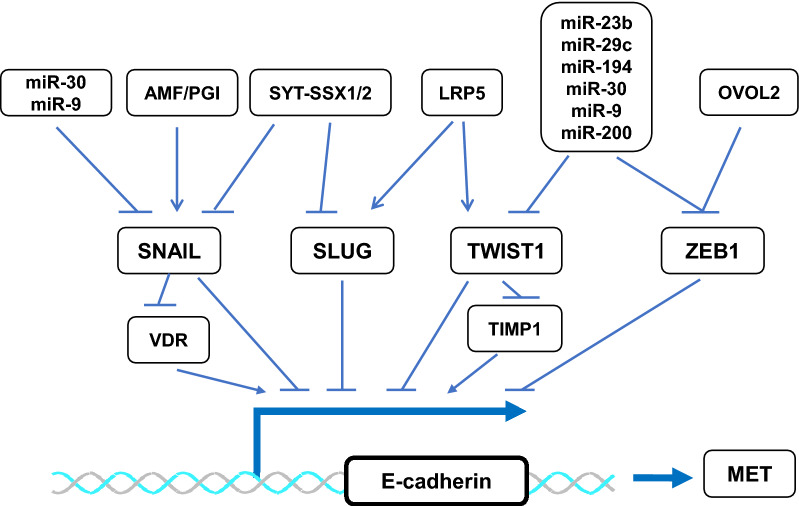
The molecular regulatory mechanisms for MET in OS cells. The EMT-inducing transcription factors including SNAIL, SLUG, TWIST1 and ZEB1 are expressed in OS cells, which directly or indirectly repress the expression of epithelial genes such as E-cadherin to maintain mesenchymal cell features. MET is initiated by inhibiting EMT-TFs through activating upstream signaling pathways such as SYT-SSX1/2, OVOL2 and miRNAs or suppressing AMF/PGI or LRP5 in OS cells. Please refer to the text for related references. *VDR* vitamin D receptor, *TIMP1* TIMP metallopeptidase inhibitor 1

Several signaling pathways can program a MET status of OS cells through regulating the transcriptional activities of EMT-TFs. These cross-talk regulatory networks are depicted in Fig. 3. Specifically, blocking Wnt/LDL receptor related protein 5 (LRP5) signaling by a soluble negative dominant form of LRP5 mutant in OS cells markedly upregulates the expression of E-cadherin, an epithelial marker, and downregulates the expression of N-cadherin, a mesenchymal marker. Inhibition of the Wnt/LRP5 signaling also downregulates the activity of EMT-TFs such as TWIST1 and SLUG [113]. Synovial sarcoma translocated-synovial sarcoma X1 and 2 (SYT-SSX1/2) interact with SNAIL and SLUG, respectively, to diminish their transcriptional repression activities on E-cadherin expression, resulting in an increase in E-cadherin expression and an acquisition of epithelial characteristics in synovial sarcoma cells [112]. In OS cells, the autocrine motility factor (AMF), also known as phosphoglucose isomerase (PGI), enhances SNAIL activity. Accordingly, silencing the expression of AMF/PGI can reduce SNAIL activity, which induces terminal differentiation of these OS cells into mature osteoblasts, resulting in suppression of the growth and pulmonary metastasis of these OS cell-derived xenografts in nude mice [114]. Ovo like zinc finger 2 (OVOL2) represses ZEB1 expression by binding to the *ZEB1* promoter, so high OVOL2 expression is associated with low ZEB1 expression in human OS. In agreement with this finding, overexpression of OVOL2 in OS cells can promote MET and suppress cell migration and invasion [115]. In addition, miRNAs can directly regulate E-cadherin expression to induce MET or indirectly regulate E-cadherin expression through targeting EMT-TFs. For example, miR-30 and miR-9 can target TWIST1, SNAIL and ZEB1 mRNAs, and miR-23b, miR-29c, miR-194 and miR-200 can downregulate TWIST1 and ZEB1 mRNAs, resulting in upregulation of E-cadherin expression [116, 117]. It is interesting to notice that, expression of one mesenchymal factor is often sufficient to induce EMT in epithelial-derived carcinomas, while expression of two epithelial factors such as GRHL2 and miR-200 are required to drive MET in human rhabdomyosarcoma cells. This may suggest that the expression of epithelial genes in mesenchymal cells requires both transcriptional de-repression (e.g. via miR-200s) and transcriptional activation (e.g. via GRHL2) events to happen [118, 119].

## Clinical implications and therapeutic opportunities of the EMT/MET plasticity of OS cells

The growing body of data on the regulation of sarcoma cell EMT/MET plasticity may offer clinical implications and therapeutic opportunities to patients. Specifically, the MET phenotype with epithelial marker expression in sarcomas may serve as prognostic markers. Withaferin-A (WFA), a naturally derived bioactive compound, is a vimentin inhibitor, which could be a promising drug against vimentin-expressing sarcoma cells. WFA treatment causes vimentin cleavage and induces sarcoma cell apoptosis. WFA also significantly inhibits growth, local recurrence and metastasis of the soft tissue sarcoma cell-derived xenografts in vivo [120]. This finding suggests that vimentin may be a good target for inhibiting soft tissue sarcomas.

EMT-TFs may also serve as potential therapeutic targets in sarcomas. It has been reported that TWIST1 is one of the 45 chemoresistant-signature genes that can predict OS patients’ response to neoadjuvant chemotherapy at the time of diagnosis. Knockdown of TWIST1 in multiple OS cell lines including HOS, SJSA-1 and 143B cells can largely overcome the chemoresistance of these cancer cells [121]. However, another study showed that TWIST1 can increase the chemosensitivity of SaOS2 and MG-63 OS cells to cisplatin treatment by downregulating endothelin-1 (ET-1) [122], because high ET-1 expression increases cell invasion and survival against cisplatin treatment in OS cells [123]. These different results from different studies might be due to the different functions of TWIST1-regulated target genes. Therefore, it may depend on specific OS cell context to determine whether TWIST1 can serve as a drug target. It has also demonstrated that OS cells that survived after cisplatin treatment at a sublethal dose exhibit a more mesenchymal phenotype and an elevated capacity to metastasize. Under these circumstances, inhibition of SNAIL can promote cisplatin sensitivity and prevent cisplatin treatment-induced EMT-like process, which results in diminished OS cell growth and survival [124]. Therefore, targeting these EMT-TFs in OS cells may help to induce MET and improve OS cell response to chemotherapy.

## Conclusion remarks

Metastasis is the major cause of cancer-related death. When compared with numerous metastasis-related researches carried out for other cancer types such as breast and prostate cancers, much fewer studies of OS metastasis have been conducted so far. OS patients treated in recent years basically still receive essentially unchanged treatments applied in 1970s [16]. These may be due to the absence of consistent genetic mutations, and the relatively low incidence and high heterogeneity of the disease. Furthermore, our limited understanding of the biology about what drives OS cell dissemination from the primary bony sites and their subsequent proliferation at a second tissue environment such as the lung may have hindered our ability to develop new therapies for treating metastatic OSs. To impact the lives of patients suffering from metastatic OSs, it will be necessary to deepen our fundamental knowledge about OS metastasis and its specific vulnerabilities at cellular and molecular levels. The cell signaling pathways implicated in OS biology through genetic and other preclinical studies mainly include PI3K/mTOR [125], TGFβ [126], RANKL/NF-κB [127], and IGF [128]. Unfortunately, clinical studies evaluating the reagents that target these pathways have yielded disappointing results. Recently, although HER2 expression has been detected in certain OSs and tested as a therapeutic target in OS, targeting HER2 with trastuzumab, an FDA approved antibody drug for breast and gastric cancers with HER2 overexpression, is still not very effective to inhibit OS growth [129]. However, immunotherapy using HER2 chimeric antigen receptor (CAR) T-cells may be developed into a promising therapeutic approach for treating HER2-positive OSs [130]. Of note, the role of HER2 in the regulation of EMT/MET plasticity in OS cells is currently unknown.

It becomes obvious that new targets still need to be identified for developing new therapeutic strategies and drugs. Further understanding of the cell plasticity in OS progression could offer new opportunities to address these issues. EMT-TFs, especially TWIST1 and SLUG, play important roles in bone development and remodeling. These EMT-TFs regulate EMT and MET plasticity of OS cells, which is not identical to their regulations in solid tumors of epithelial origin [98]. Further investigation and deeper understanding of the EMT/MET-regulatory machineries in OS cells may help to identify druggable molecular targets. Furthermore, given the complexity of EMT/MET-like regulatory networks and the ability of cancer cells to adapt to stress conditions, targeting one protein or pathway may not be sufficient to completely impede EMT-related process or initiate MET. The future druggable targets that can be identified from the EMT/MET-like regulatory networks for treating metastasis of OS cells may be used in combination with the current surgery and chemotherapy treatments to achieve better clinical outcomes.

## Data Availability

Not applicable.

## Abbreviations

AMF: Autocrine motility factor
CAM: Chick chorioallantoic membrane
EMT: Epithelial-to-mesenchymal transition
EMT-TF: Epithelial-to-mesenchymal transition transcription factor
ES/PNET: Ewing sarcoma/primitive neuroectodermal tumor
ET-1: Downregulating endothelin-1
GEM: Genetically engineered mouse
LRP5: LDL receptor related protein 5
MET: Mesenchymal-to-epithelial transition
Meta: Metastasis
OB-cad: Adhesion molecule osteoblast cadherin
OS: Osteosarcoma
OVOL2: Ovo like zinc finger 2
PDX: Patient-derived xenograft
PGI: Phosphoglucose isomerase
SYT-SSX1/2: Synovial sarcoma translocated-synovial sarcoma X 1/2
TIMP1: TIMP metallopeptidase inhibitor 1
VDR: Vitamin D receptor
WFA: Withaferin-A
